# Stu-miR827-Targeted *StWRKY48* Transcription Factor Negatively Regulates Drought Tolerance of Potato by Increasing Leaf Stomatal Density

**DOI:** 10.3390/ijms232314805

**Published:** 2022-11-26

**Authors:** Jiangwei Yang, Ning Zhang, Jiangping Bai, Xiaoqin Duan, Luhe Zhang, Shengyan Liu, Xun Tang, Xin Jin, Shigui Li, Huaijun Si

**Affiliations:** 1 State Key Laboratory of Aridland Crop Science, Gansu Agricultural University, Lanzhou 730070, China; 2 College of Life Science and Technology, Gansu Agricultural University, Lanzhou 730070, China; 3 College of Agronomy, Gansu Agricultural University, Lanzhou 730070, China; 4 College of Forestry, Gansu Agricultural University, Lanzhou 730070, China

**Keywords:** Stu-miR827, *StWRKY48*, stomatal density, drought stress, *Solanum tuberosum*

## Abstract

Stomata are specialized portals in plant leaves to modulate water loss from plants to the atmosphere by control of the transpiration, thereby determining the water-use efficiency and drought resistance of plants. Despite that the stomata developmental progression is well-understood at the molecular level, the experimental evidence that miRNA regulates stomata development is still lacking, and the underlying mechanism remains elusive. This study demonstrates the involvement of stu-miR827 in regulating the drought tolerance of potato due to its control over the leaf stomatal density. The expression analysis showed that stu-miR827 was obviously repressed by drought stresses and then rapidly increased after rewatering. Suppressing the expression of stu-miR827 transgenic potato lines showed an increase in stomatal density, correlating with a weaker drought resistance compared with wildtype potato lines. In addition, *StWRKY48* was identified as the target gene of stu-miR827, and the expression of *StWRKY48* was obviously induced by drought stresses and was greatly upregulated in stu-miR827 knockdown transgenic potato lines, suggesting its involvement in the drought stress response. Importantly, the expression of genes associated with stomata development, such as *SDD* (stomatal density and distribution) and *TMM* (too many mouths), was seriously suppressed in transgenic lines. Altogether, these observations demonstrated that suppression of stu-miR827 might lead to overexpression of *StWRKY48*, which may contribute to negatively regulating the drought adaptation of potato by increasing the stomatal density. The results may facilitate functional studies of miRNAs in the process of drought tolerance in plants.

## 1. Introduction

Drought stress is one of the most important environmental factors restricting the productivity and distribution of crops [1,2]. Plants have evolved a wide variety of inducible defense mechanisms in response to drought stress. An effective strategy of drought resistance used by plants is to reduce the transpirational water loss from leaves by controlling the stomatal density and stomatal conductance. The stoma is a main channel that regulates gas exchange and water release between the environment and the plant body [3] and is differentiated from protodermal cells. Molecular genetics research has revealed that the processes of stomatal differentiation are regulated by a set of genes, such as speechless (*SPCH*), arrested meristemoids (*MUTE*), *FAMA*, stomatal density and distribution (*SDD*), too many mouths (*TMM*), epidermal patterning factor-like (*EPFL*), ERECTA (*ER*), ERECTA-LIKE 1 (*ERL1*), *ERL2*, and YODA (*YDA*), which are known to participate in stomatal development [4,5,6]. Transcriptional regulation is the most important regulatory mechanism for gene expression, and studies have showed that transcription factors (TFs) also regulated stomatal development [5,7]. Yoo et al. showed that GTL1 TFs regulated drought tolerance by modulating stomatal density in *Arabidopsis* [8]. *AtEDT1*/*HDG11* as START TFs modulated stomatal density by activating *ERECTA* and *E2Fa* [9]. However, the mechanisms by which WRKY TFs regulate stomatal development are still largely unknown in potato.

The WRKY TF superfamily is one of the largest plant-specific transcription factors and contains numerous members, which can bind sequence-specifically to the DNA sequence motif: (T)(T)TGAC(C/T) (W-box), in the promoter regions of target genes [10], and hence can regulate the transcription levels of target genes to perform their functions [11]. Since the first WRKY gene was discovered in sweet potato [12], numerous WRKY proteins have been identified from other plants, with an ever-increasing number of functions in essential physiological and developmental processes [13]. Members of some WRKY genes have been found to play roles in plant responses to various abiotic stresses [14]. There are two WRKYs (*AtWRKY25* and *AtWRKY33*) which have been extensively studied in *Arabidopsis*. Available data have shown that the *AtWRKY25* and *AtWRKY33* cascade was activated by salt stress and plays a role in increasing salt tolerance in *Arabidopsis* [15]. Overexpression of soybean *GmWRKY54* and *GmWRKY13* in transgenic *Arabidopsis* resulted in greater salt and drought tolerance than in wildtype plants [16]. *OsWRKY11* and *OsWRKY45* from rice have been demonstrated to play a regulatory role in salt tolerance [17,18]. Finally, overexpression of *TaWRKY10* from wheat was reported to result in enhanced drought and salt tolerance in transgenic tobacco plants [19]. However, the knowledge of the post-transcriptional regulation of WRKY genes is limited.

MiRNAs are 21–22-nucleotide small RNAs that have been known to play vital regulatory roles in various functions in plants by complementarily binding to their target gene sites, and inhibiting target gene expression [20]. Until now, numerous miRNAs were found, and majority of their target genes were demonstrated to encode TFs that serve as important regulators of plant development and responses to various environmental stimuli [21]. For example, the miR156 has been reported to regulate the transitions of vegetative-to-reproductive and juvenile-to-adult by targeting SPL transcription factors [22]. In *Arabidopsis,* miR472 targeted NBS-LRR genes that exhibit an immune response [23]. The miR159 targets MYB transcription factors that inhibit plant growth and promote programmed cell death in *Arabidopsis* [24]. miR160 targets ARF transcription factor and inhibits the development of the symbiotic nodule in soybean [25]. miR164 targets NAC transcription factors to regulate plant lateral root development and negatively regulate drought resistance [26]. Although an accumulating body of research has also shown that miRNAs are involved in regulating multiple biotic/abiotic stress responses, the main research results were found in model plants [27,28,29,30,31,32,33,34,35]. Nevertheless, the regulating mechanism of WRKY genes by miRNA is rarely reported in potato. In this study, a potato miRNA (stu-miR827-5p) was predicted and confirmed targeting *StWRKY48*. Additionally, suppressing the expression of stu-miR827-5p could regulate overexpression of *StWRKY48* to reduce the drought resistance of potato through increasing stomatal density. These results revealed significant roles of miRNAs in the regulation of potato drought resistance, as well as provided a valuable resource for drought tolerance breeding in other food crops.

## 2. Results

### 2.1. Identification, Characterization, and Expression Analysis of stu-miR827-5p

**Figure 1:**
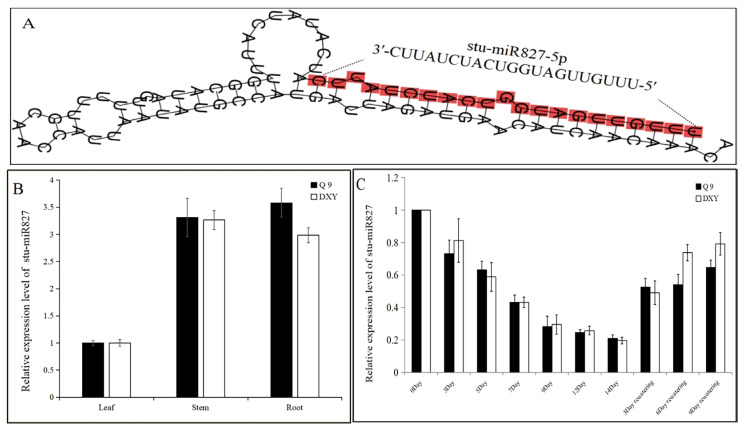
Characterization and qRT-qPCR analyses of stu-miR827-5p expression profiles in different tissues and response to drought stress in potato. (**A**) Hairpin secondary structures of stu-miR827 precursor as predicted by MFold, and the mature stu-miR827-5p sequence is highlighted in red. (**B**) Relative expression levels of the stu-miR827-5p in different tissues of potato. (**C**) Relative expression levels of the stu-miR827-5p under drought stress. Q9 represents the potato variety “Qingshu9” and DXY represents the potato variety “Atlantic”.

Stu-miR827 was firstly reported in 2013 and obtained by small-RNA sequencing [36]. The 87 nt lengths of the stu-miR827 precursor can be folded in a stem–loop structure (Figure 1A), which is the typical hairpin structure of the miRNAs, and the 21 nt lengths of the mature sequence within the 5′arm of the stem–loop structure. The detailed information of stu-miR827 is shown in Table 1. Previous studies have shown miR827 responses to abiotic stress in plants [37,38]. To determine whether stu-miR827-5p plays roles in the potato drought stress response, quantitative real-time PCR (qRT-PCR) was used to measure the expression levels of stu-miR827 in different tissues (leaves, stems, and roots) and the response to drought stresses in potato. The results indicate that stu-miR827 was expressed at obviously low levels in potato leaves compared with stems and roots (Figure 1B) and was significantly repressed by drought stresses and induced after rewatering (Figure 1C). These results suggested a potential role of stu-miR827 in potato leaves for responding to drought stress.

### 2.2. Generation of Transgenic Potato with Repressed stu-miR827-5p Expression by STTM Approach

**Figure 2:**
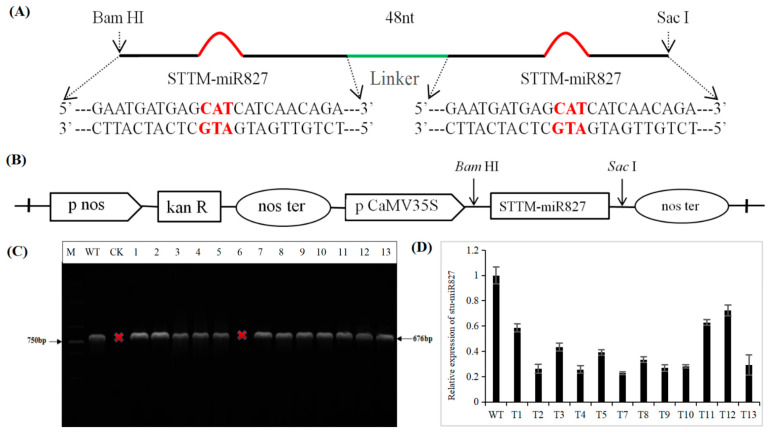
Construction of STTM-miR827-5p vector and potato transformation. (**A**) The short tandem target mimic (STTM) construct used for silencing stu-miR827-5p. (**B**) The STTM-miR827-5p fragment was cloned into the pBI121 binary vector under the CaMV 35S promoter. (**C**) PCR detection of the *NPTII* gene in transgenic plants. Note: M: DL2000 marker; WT: wildtype plants transformed with the empty vector (pBI121); CK: wildtype plants; 1–13: transformed plant lines containing the STTM-miR827-5p fragment. (**D**) Expression levels of miR827a-5p in WT and transgenic lines.

Previous articles and studies have demonstrated that short tandem target mimic (STTM) is an effective technique for counteracting the activity of miRNAs in model plants [39,40]. In this study, to inhibit stu-miR827-5p activity and investigate the function of stu-miR827 in potato responses to drought stress, we designed and constructed the STTM827-5p configuration, which has two miRNA target mimic sequences, separated by a 48-nucleotide spacer. Each of the target mimic sequences were non-cleavable miR827-5p-binding sites and had a bulge containing three additional nucleotides (CTA) (Figure 2A), which have complementarities suitable to bind the mature sequence of stu-miR827-5p. The STTM827-5p fragment was firstly inserted in the T-19 vector and cloned into the pBI121 vector under the CaMV 35S promoter (Figure 2B). Then, the recombined vector was introduced into potato cv. “Desiree” by the Agrobacterium-mediated cotyledon transformation method and obtained 13 independent potato transgenic plants, which were screened by genomic DNA-PCR detection with specific primers of the *NPTII* gene (Figure 2C). To further evaluate the transgenic lines, qRT-PCR was used to analyze the expressions of stu-miR827-5p. The results showed that all the transgenic lines had a lower expression of stu-miR827-5p (Figure 2D).

### 2.3. Suppression of microRNA827-5p Increases Stomatal Density

**Figure 3:**
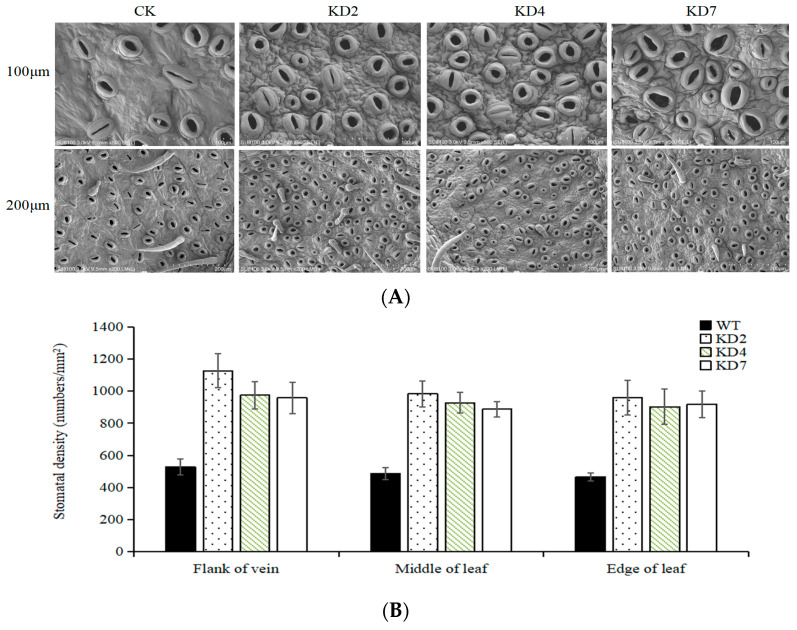
Leaf stomatal density and expression of stomatal development genes in transgenic and WT plants. (**A**) Scanning electron micrograph of the abaxial leaf epidermis. (**B**) Leaf stomatal density for transgenic and WT plants.

Stomatal density in plant leaves is functionally associated with water-use efficiency and drought tolerance of plants. Stomatal densities in different regions of the leaf (leaf edge, leaf vein-flanking region, and the area between the two) were comparatively analyzed both in transgenic (DK2, DK4, and DK7) and WT plants. Test results indicate that stomatal density in transgenic lines was obviously increased compared to WT (Figure 3A), and the vein-flanking region of leaves had a higher average stomatal density than other regions (Figure 3B). These results implicate that suppression of stu-miR827-5p increases the stomatal density of transgenic potato.

### 2.4. Suppression of microRNA827-5p Reduced Drought Tolerance

**Figure 4:**
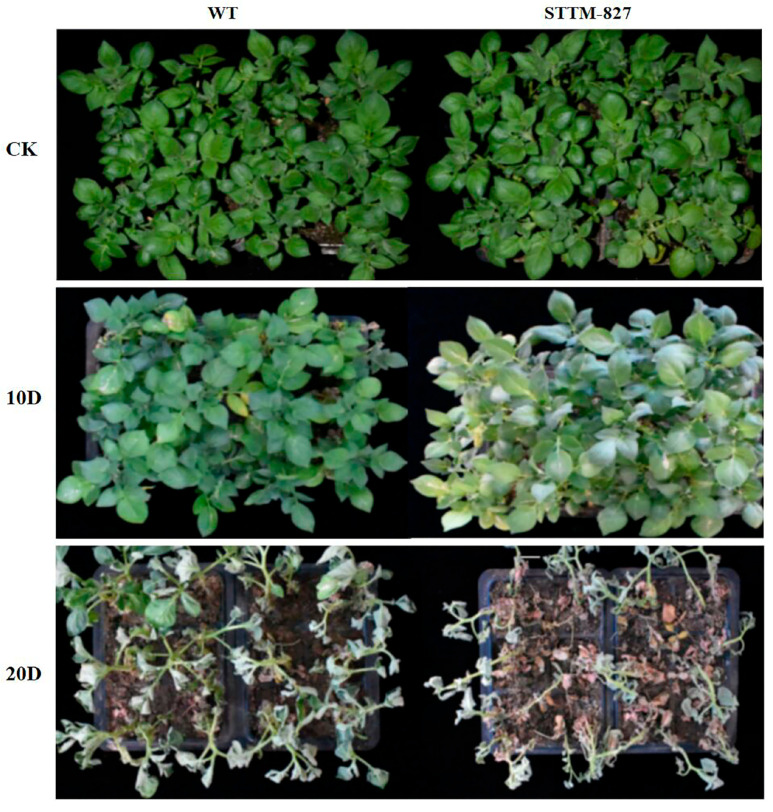
Drought resistance of transgenic potato plants stably overexpressing STTM-miR827-5p fragment. WT: wide potato plants; STTM-827: transgenic potato plants stably overexpressing STTM-miR827-5p fragment; CK: WT and transgenic plants without drought treatment; 10D: the drought symptoms of WT and transgenic plants after the 10th day of drought treatment; 20D: the drought symptoms of WT and the transgenic plants after the 20th day of drought treatment.

To investigate the role that stu-miR827 plays in the potato drought stress response, the fully developed transgenic plant lines and WT plants were subjected to a 20-day drought treatment period. The drought symptoms were visible for the whole plant of the transgenic plant lines and WT, but the transgenic plant lines displayed a more pronounced phenotype than the CK plants (Figure 4). The wilting phenotype and tissue damage were significantly more observed in transgenic plants than in the CK plants. The drought-elicited damage was more pronounced in stu-miR827-5p STTM plants, indicating a reduced tolerance to drought stress in miR827 knockdown transgenic potato plants.

### 2.5. Suppression of microRNA827-5p Altered Drought-Relevant Physiological Indexes

**Figure 5:**
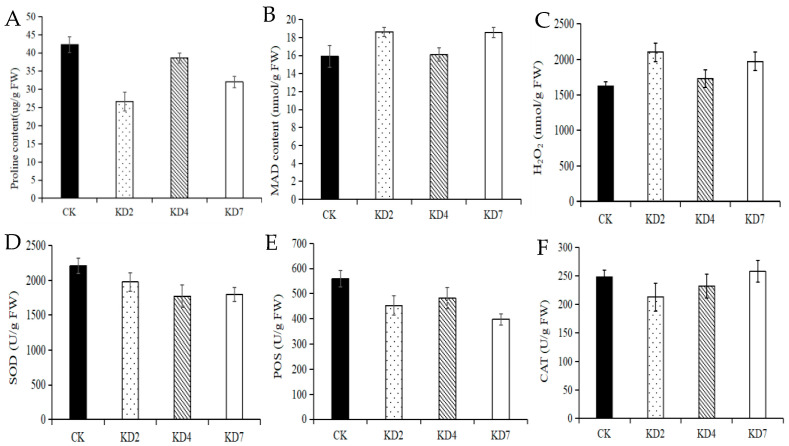
The drought-relevant physiological indexes between transgenic potato lines and CK. (**A**) The content of proline, (**B**) the content of MDA, (**C**) the content of H_2_O_2_, (**D**) the enzyme activity of SOD, (**E**) the enzyme activity of POD, and (**F**) the enzyme activity of CAT.

Three transgenic plant lines (L2, L4, and L7) were used to determine drought-related physiological indexes, including content of proline, MDA, and H_2_O_2_, and the enzyme activity of POD, CAT, and SOD. The results showed that suppression of microRNA827-5p contributed to the decreased content of proline (Figure 5A), as well as the enzyme activity of POD (Figure 5B) and SOD (Figure 5C). However, the contents of MDA (Figure 5D) and H_2_O_2_ (Figure 5E) were slightly increased in three transgenic potato lines. The change of CAT enzyme activity was not obvious between the transgenic lines and wild potato plant: the enzyme activity of CAT was decreased in KD2 and KD4, and increased in KD7 (Figure 5F).

### 2.6. Identification of stu-miR827-5p-Targeted StWRKY48 Gene

**Figure 6:**
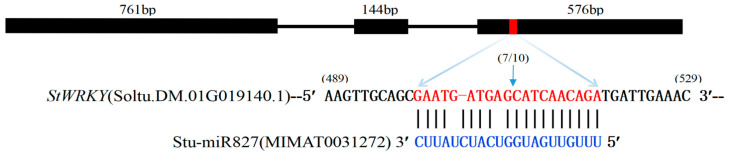
RLM-5′RACE verification of target mRNA cleavage sites generated by stu-miR827. Fractions within parentheses are the proportions of 5′RACE clones showing these cleavage sites out of all sequenced clones.

To understand the underlying molecular mechanisms of stu-miR827-mediated plant responses to drought stress, we sought to identify putative targets of stu-miR827-5p in potato. A plant small-RNA target analysis tool (psRNATarget: http://plantgrn.noble.org/psRNATarget/) (2017 Update) was applied to predict targets in the potato genome, and a potato transcript sequence (Soltu.DM.01G019140.1) was predicted as the candidate target of stu-miR827-3p (Table 2). It was obtained from the potato genome database and was named as *StWRKY48* based on the best homologous genes in *Arabidopsis* and tomato. The full-length cDNA sequence of *StWRKY48* was 1481 bp, with a 166 bp length of 5’ UTR (untranslated region) and 295 bp length of 3’ UTR, which contained a 1020 bp open reading frame (ORF) encoding a protein of 339 amino acids. It predicted that the relative molecular mass was 67.18 kDa, and the isoelectric point (pI) was 9.22. The detailed information is shown in Table S1. Additionally, to further determine the stu-miR827-mediated cleavage sites in *StWRKY48* mRNA, a widely used RLM-5′RACE assay was performed. Our results showed that the cleaved site of *StWRKY48* mRNA was between the 509th and 510th nucleotides, which were located at the 10th/11th nucleotides of the stu-miR827-5p/*StWRKY48* mRNA complementary region (Figure 6), demonstrating that *StWRKY48* were targeted by stu-miR827-5p in vivo.

### 2.7. Subcellular Localization and Expression Pattern of StWRKY48

**Figure 7:**
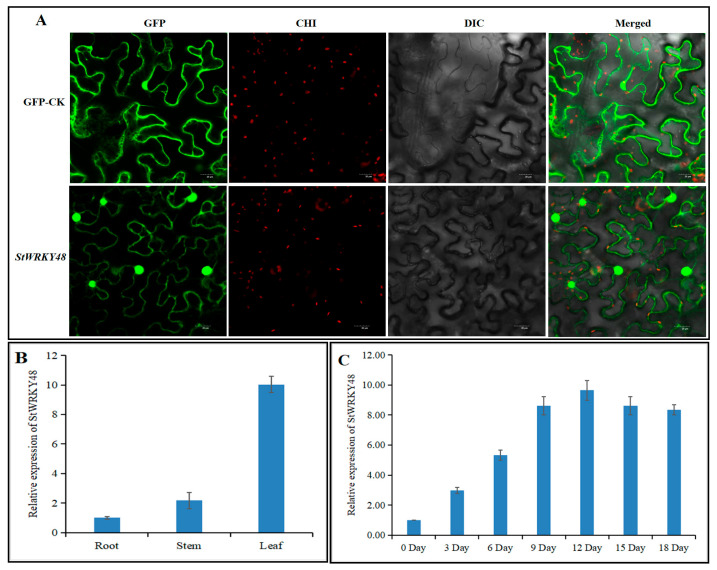
Subcellular localization, tissue-specific, and drought stress expression profiles of *StWRKY48*. (**A**) Subcellular localization of StWRKY48-GFP and GFP genes, 35S::StWRKY48-GFP and 35S::GFP control vectors were transiently expressed in tobacco leaf. (**B**) Expressional analysis of *StWRKY48* in the root, stem, and leaf of potato variety “Atlantic”. (**C**) RT-qPCR analyses of *StWRKY48* expression profiles in response to drought stress in potato variety “Atlantic” leaves.

To explore the function of stu-miR827-targeted *StWRKY48*, the subcellular localization was analyzed. The result showed that the green fluorescent signal was distributed throughout the nucleus (Figure 7A). Thus, *StWRKY48* was localized to the nucleus. Tissue-specific expression of the *StWRKY48* gene was performed using three different potato tissues (root, stem, and leaf). The results showed that *StWRKY48* had a high level of expression in potato leaves (Figure 7B). This suggests that *StWRKY48* may play a role in potato leaves. To verify whether stu-miR827-5p-targeted *StWRKY48* was involved in the response to drought stress, RT-qPCR was performed to examine the expression of the *StWRKY48* transcript under drought stress. The result showed that *StWRKY48* was significantly induced under drought stress (Figure 7C).

### 2.8. Knockdown of stu-miR827-5p Increases the Levels of StWRKY48

**Figure 8:**
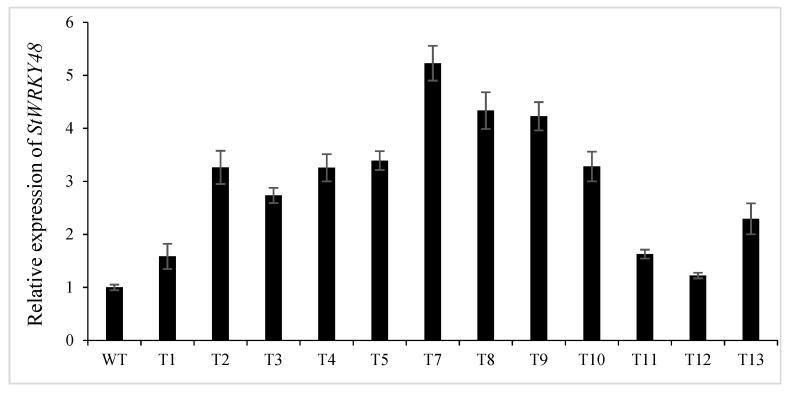
Expression levels of *StWRKY48* in the transgenic lines. CK: wildtype plant; T1–T13: transgenic lines.

To evaluate whether STTM827 affected stu-miR827 activity, the expression levels of stu-miR827-5p and its target gene *StWRKY48* were determined in transgenic plant lines and control plants by RT-qPCR. As expected, the expression level of miR827-5p was efficiently decreased in the transgenic lines compared to CK (Figure 2D), while the transcript levels of *StWRKY48* in transgenic lines were consequently increased in independent transgenic plants (Figure 8), with the expression levels of *StWRKY48* in transgenic lines T2, T4, T5, T7, T8, T9, and T10 being more than 3-fold higher than those of the control. These results suggested that suppression of stu-miR827-5p can increase the expression levels of *StWRKY48*.

### 2.9. Suppression of microRNA827-5p Affects the Expression of Stomata Development-Related Genes

**Figure 9:**
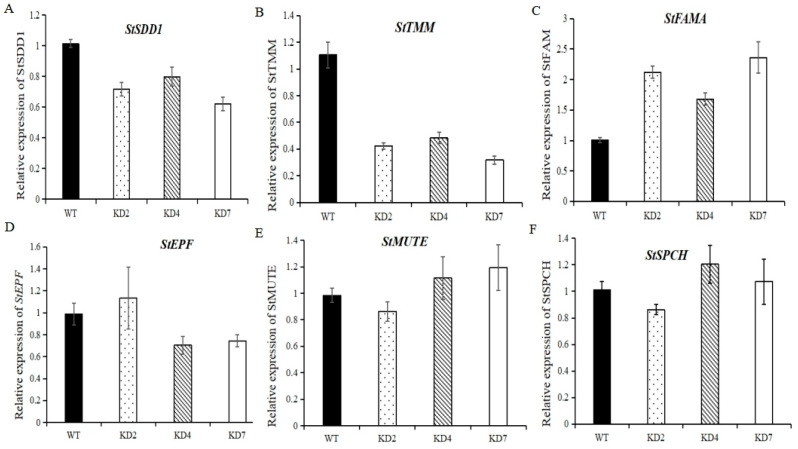
Expression of stomatal development-related genes in transgenic and WT plants. (**A**–**F**) Expression of the stomatal development genes *StSDD1* (**A**), *StTMM* (**B**), *StFAMA* (**C**), *StEPF* (**D**), *StMUTE* (**E**), and *StSPCH* (**F**).

To further investigate the function of stu-miR827-5p for potato stomatal density, we searched for and obtained six stomata development-related genes from the potato genome, including *StSDD* (stomatal density and distribution, Soltu.DM.07G004760.1), *StTMM* (too many mouths, Soltu.DM.03 G016170.1), *StEPF* (epidermal patterning factor, Soltu.DM.12G028300.1), *StSPCH* (SPEECHLESS, Soltu.DM.03G022880.1), *StMUTE* (Soltu.DM.01G024030.1), and *StFAMA* (Soltu.DM.05G023840.1). We subsequently analyzed the expression levels of those six genes between transgenic and wildtype plants, and a significant downregulation of *StSDD1* and *StTMM* genes was detected in potato transgenic lines (Figure 9A,B). We also observed that the expression of *StFAMA* was obviously increased in three transgenic lines compared to wildtype plants (Figure 9C), while the expression level of *StEPF* was obviously suppressed in KD4 and KD7 and was slightly elevated in KD2 (Figure 9D). In addition, only subtle changes were observed for *StMUTE* and *StSPCH* genes between the transgenic and wildtype plants, the expressional levels of *StMUTE* and *StSPCH* were slightly lower in line KD2 and remained unchanged in lines KD4 and KD7 (Figure 9E,F). All things considered, *StWRKY48* positively regulates stomatal density, mainly by downregulating *StSDD1* and *StTMM* genes.

## 3. Discussion

### 3.1. Stu-miR827-Mediated StWRKY48 mRNA Cleavage In Vivo

The WRKY TFs family is one of the largest and most typical transcription factors in the plant kingdom. Numerous WRKY TFs are found as key regulators in many plant-specific processes, including growth and development [41,42], signal transduction [43], and responses to various biotic [17] and abiotic stresses [15]. However, little information is known about the post-transcriptional regulation mechanism of WRKY TFs in plants. MiRNAs are a class of endogenous, non-coding, small-molecule RNAs that play crucial roles in many plant processes [21,44,45,46,47]. Herein, we identified a drought-responsive miRNA (stu-miR827), and target prediction indicated that its predicted target gene is a WRKY TF family member (*StWRKY48*), and cleavage positions of *StWRKY48* were also effectively validated by the 5′RLM-RACE assay. Quantitative PCR assays also showed that the expression levels of stu-miR827-5p and *StWRKY48* exhibited an opposite trend under drought stress, which was similar to that previously reported for the expression pattern between miRNAs and target genes [48]. In addition, suppressed expression of stu-miR827-5p transgenic lines was obtained by STTM, and suppressed expression of stu-miR827-5p can lead to overexpression of *StWRKY48*. Collectively, we demonstrated that *StWRKY48* is the target gene of stu-miR827 and is cleaved by stu-miR827 in vivo.

### 3.2. Stu-miR827 Negatively Regulates Drought Tolerance in Potato

As key regulators, increasing evidence have been widely suggested that plant miRNAs and their target genes play important roles in the plant response to various environmental stresses, and an increasing number of stress-induced miRNAs and their target genes have been identified in both model and crop plants [49,50,51,52]. For instance, the target genes of miR159 encoded MYB family TFs [51,53], which are widely involved in various stress responses. Previous studies have shown that a low abundance of miR159 could contribute to the accumulation of two MYB TFs (*Os-MYB4* and *Os-MYB3R-2*), and accumulation of the cold-regulated rice MYB TFs can help plants to enhance the freezing tolerance [48,52,54]. The target gene of miR398 is encoding copper/zinc superoxide dismutase (Cu/Zn-SOD, CSD), that was a scavenger enzyme of ROS (reactive oxygen species) [55]. Downregulation of miR398 resulted in an increase in CSD expression and tolerance to oxidative stress [56]. The miR395 was downregulated under drought stress and the target gene of miR395 is ATP sulfurylase (APS) [57], which is a ubiquitous enzyme that catalyzes the primary step of the intracellular response to environmental stress, and overexpression of APS protein can help plants to enrich the content of glutathione and increase the stress tolerance [49]. The miR399 response to Pi starvation and the target gene of miR399 is *UBC24* (ubiquitin-conjugation E2 enzyme), and the expression pattern of miR399 and its target gene *UBC24* were negatively correlated under Pi starvation: miR399 was upregulated and *UBC24* was downregulated in *Arabidopsis* [50]. Increasing research has shown that WRKY TFs play a paramount role in plant responses to various abiotic stresses [15,16,17,19]. However, little is known concerning the mechanism of miRNA regulating WRKY genes in plants.

Herein, the stu-miR827-targeted *StWRKY48* was identified by bioinformatics analysis and RNA ligase-mediated 5′RACE (5′RLM-RACE) (Figure 2). The expression relations of miRNA and its target are usually used as an indicator for the research function of miRNAs. The expression pattern of stu-miR827 and *StWRKY48* under drought stress in potato was also examined. Results indicated that the expression level of stu-miR827 was downregulated with drought stress (Figure 1), but the expression level of *StWRKY48* was upregulated (Figure 3). In addition, loss-of-function of the stu-miR827 mutant was also obtained by STTM in potato. The RT-qPCR assay showed that stu-miR827-5p expression was significantly decreased, while *StWRKY48* greatly increased in transgenic lines. Noteworthily, the transgenic potato lines showed a reduced tolerance to drought stress, similar to previous reports in *Arabidopsis* and rice. In *Arabidopsis*, 18 *AtWRKY* genes were induced by salt stress and 4 *AtWRKY* genes were reported to regulate the drought response [58]. Overexpression of *AtWRKY25* or *AtWRKY33* can enhance the salt tolerance of *Arabidopsis* [15], while overexpression of *AtWRKY18* or *AtWRKY60* increased the sensitivity of the plant to salt stress [59]. *AtWRKY54* and *AtWRKY70* regulate osmotic stress by working as negative regulators of stomata closure, and the double mutants exhibited clearly enhanced tolerance to osmotic stress [60]. *AtWRKY57* can elevate the levels of ABA to improve drought tolerance [61]. In rice, expression of 27 WRKY genes was induced in response to salt stress, of which 26 were upregulated [62]. In transgenic rice, overexpression of *OsWRKY11* and *OsWRKY45* resulted in enhanced salt and drought tolerance [17,18]. The grapevine *VvWRKY11* gene is involved in the response to dehydration stress, and overexpression of this gene in *Arabidopsis* led to greater tolerance to drought stress induced by mannitol compared to wildtype plants [63]. With *VvWRKY11*, the transgenic *Arabidopsis* lines overexpressing *GsWRKY20* also showed enhanced drought tolerance, while transgenic *Arabidopsis* plants overexpressing the soybean *GmWRKY54* gene were more salt- and drought-tolerant than the control, and *GmWRKY13* overexpression resulted in increased sensitivity to salt and mannitol stress [16]. Collectively, the data indicated that suppressed expression of stu-miR827-5p improved the drought tolerance of potato by leading to overexpression of *StWRKY48*. These results indicated that decreased expression of stu-miR827-5p might drive overexpression of *StMAPK48*, which may contribute to regulate the drought adaptation of potato plants.

### 3.3. Stu-miR827 Positively Regulates Stomatal Density by Suppressing the Expression of StSDD1 and StTMM Genes

**Figure 10:**
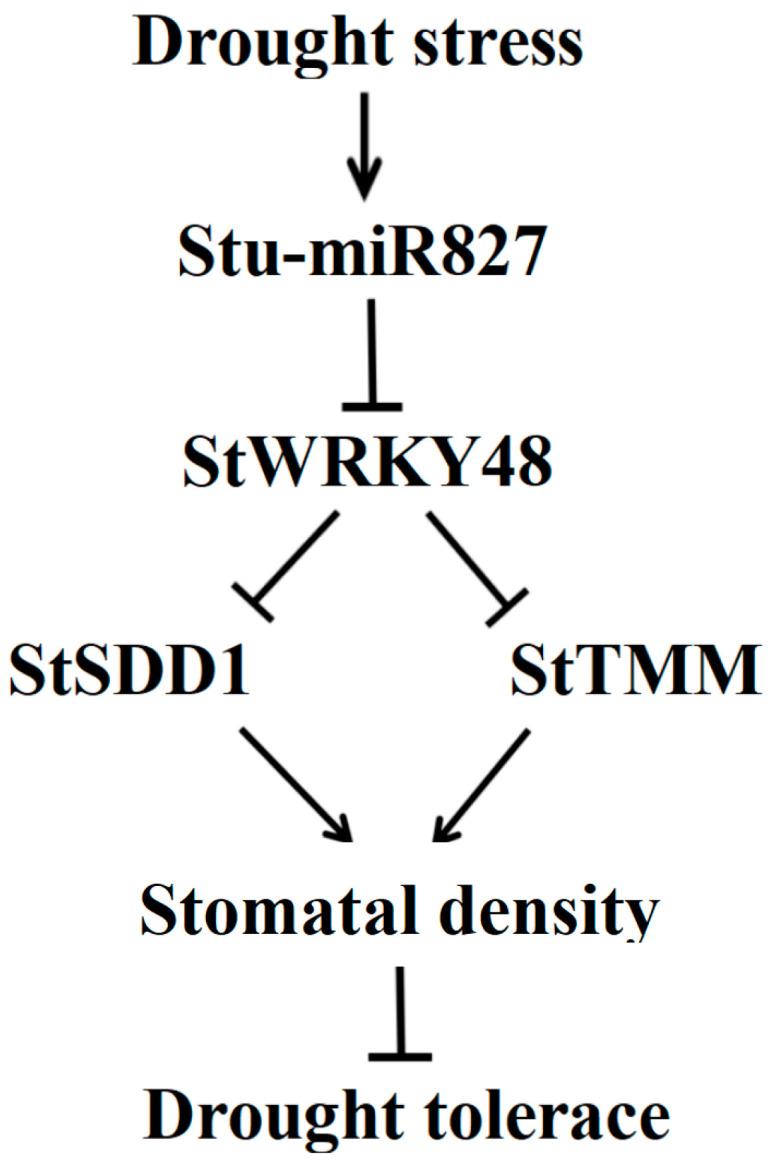
Schematic of stu-miR827 response in potato upon drought stress by regulating stomatal density.

Plant drought tolerance is mainly determined by water loss through the stomata [64]. Stomatal development and differentiation are regulated by a number of genes, including *SPCH*, *MUTE*, *FAMA*, *SDD1*, *TMM*, *EPFL*, *ERECTA*, *ERL1*, *ERL2*, *YODA*, and others, with *SDD1* and *TMM* acting as negative regulators of stomatal distribution and density [4]. SDD1, a subtilisin-like serine protease, participates in TMM-ER signaling pathways, most likely by catalyzing the conversion of pro-peptides into ligands, and reduces drought resistance by increasing stomatal density [65]. Previous research on modifying *SDD1* expression levels have demonstrated changes in stomatal density [66]. In *Arabidopsis*, *sdd* mutants increase the leaf stomatal number by two- to four-fold [67]. *TMM* encodes receptor-like protein, which is an indispensable component of the ERf signaling pathway in stomatal development [68,69], complexed with ERECTA family receptor kinases to specifically restrict stomatal patterning and density, and is only found in land plants [4,70,71]. In this study, suppression of stu-miR827 in potato led to a significant increase in leaf stomatal density compared to WT plants. In addition, the transcript levels of *StSDD1* and *StTMM* genes were significantly downregulated in transgenic lines of suppressing stu-miR827 compared to WT plants. These results suggested that stu-miR827 increased stomatal density mainly via transcriptional repression of *SDD1* and *TMM* genes. We also analyzed the expression levels of the stu-miR827-targeted *StWRKY48* gene between transgenic lines and wildtype plants, and the results showed that *StWRKY48* was largely increased in stu-miR827 suppressed in transgenic lines. This implied that *SDD1* and *TMM* were possibly the target genes of *StWRKY48*. Further experiments are therefore needed to confirm the regulatory mechanism between *StWRKY48* and *StSDD1* and *StTMM* genes. Interestingly, A recent study found that overexpression of *VvWRKY18* in *Arabidopsis* increased stomatal density by regulating the expression of *AtSDD1* and *AtTMM* genes [72]. Therefore, our findings, along with those of others, showed that suppressing stu-miR827 led to overexpression of *StWRKY48*, which primarily reduced the expression of *StSDD1* and *StTMM* genes and increased the number of stomata on the epiderm of the potato leaf, improving the rate of leaf water loss and, as a result, reducing drought tolerance (Figure 10). These findings open up a new window into the function of miRNAs in the regulation of stomatal density and distribution in plants, allowing for further research into the role of miRNAs in plant adaptation to drought stress.

## 4. Methods

### 4.1. The Quantitative Real-Time PCR (qRT-PCR) Analysis

For stu-miR827 quantification, total RNA was extracted from potato leaf samples using the TRIzol Reagent (Invitrogen, Carlsbad, CA, USA), and a tail was added to the 3′ end of RNA. The reverse transcription was performed using the miRcute miRNA First-Strand cDNA Synthesis Kit (Tiangen, Beijing, China) according to the manufacturer’s instructions. *St18SRNA* was used as an internal reference gene. The gene-specific primer of stu-miR827 and internal reference primers are reported in Table 3. For *StWRKY48*, *StSDD*, *StTMM*, *StEPF*, *StSPCH*, *StMUTE,* and *StFAMA* genes’ expression analysis, total RNA was isolated using TRIzol reagent following the manufacturer’s instructions (Invitrogen, Carlsbad, CA, USA). The quality and purity of each RNA sample were tested using an IMPLEN Nanophotometer (LIFE, Germang), and the integrity of the RNA sample was checked by resolution on a 1.5% agarose gel. The quantitative real-time PCR (qRT-PCR) was performed by SYBR Green I (Takara, Japan). The potato *EF1a* gene was used as the internal reference [73]. In the study, the RT-qPCR was carried out using the Super Real PreMix Plus Kit (SYBR Green, Tiangen) in the Roche LightCycler 96 Real-Time PCR System, and all primers used for qRT-PCR analysis are shown in Table 3. At least three independent biological samples were subjected to a minimum of three technical replicates for RT-qPCR analysis. The relative expression changes were calculated using the 2^-△△Ct^ method based on the RT-qPCR data.

### 4.2. Construction of Silencing Plasmid of stu-miR827-5p

To obtain miR827-5p silent potato mutants, STTM (short tandem target mimic) was used to construct the silencing plasmid of stu-miR827-5p based on the previously described method [74]. The construct contains the two copies of mimic target sites, and they were linked by a 48-nucleotide linker. Each mimic target area has an imperfect stu-miR827-5p binding site in the targeted gene (*StWRKY48*) and contains a hump of three additional nucleotides (CTA) in the middle of the stu-miR827-5p binding site. A 96-nucleotide STTM-miR827-5p digested fragment was cloned into the pBI121 binary vector and named pBI121-STTM827. The recombinant plasmid (pBI121-STTM827) was introduced into *Agrobacterium tumefaciens* strain LBA4404 for plant transformation, which can compete with the target gene to combine with the mature sequence of stu-miR827-5p to reduce the activity of stu-miR827-5p in vivo.

### 4.3. Transformation of Potato and Identification of Transgenic Lines

For potato transformation, four-week-old potato test-tube plants of variety “Desiree” were used as the experimental material for Agrobacterium-mediated transformation according to the previously reported method [75]. Putative miR827-5p knockdown transgenic potato lines were selected from kanamycin-resistant plants. Then, propagating the kanamycin-resistant plants in a growth chamber for further molecular validation, the presence of the transferred neomycin phosphotransferase II (*NPTII*) gene in the regenerating plantlets was demonstrated using PCR with gene-specific primers (forward primer: 5′-GCTATGACTGGGCACAACAG-3′; reverse primer: 5′-ATACCGTAAAGCACGAGG AA-3’), and the expected amplified product was 676 bp. The abundance of stu-miR827-5p in the selected silencing lines was examined by RT-qPCR.

### 4.4. Analysis of Transgenic Potatoes’ Resistance to Drought Stress

To further understand the drought performance of stu-miR827 in potato, the stu-miR827 knockdown transgenic lines and the WT test-tube plantlets were transferred from the nursery site and grown in a greenhouse of Gansu Agricultural University under conditions of 22 °C/26 °C (night/day) and a photoperiod of 16/8 h (day/night). For drought assays, water was withheld from three-week-old plants grown in soil. Photographs of plants were taken with a digital camera (Nikon D3000, Tokyo, Japan), and then the plants were harvested for further analysis.

### 4.5. Measurement of Reactive Oxygen Species (ROS), Proline, and Malonaldehyde (MDA) Levels and Antioxidant Enzyme Activity

Here, 21-day-old transgenic plantlets and WT after 10 days of drought treatment were collected to measure relevant physiological indexes. The leaves of each specimen were collected and used to determine levels of proline and malonaldehyde (MDA), and the enzyme activity of peroxidase (POD), catalase (CAT), and superoxide dismutase (SOD). The ROS levels [76], proline content [77], and MDA concentration [78] were measured according to previous methods. The enzyme activity of POD, SOD, and CAT was detected according to the method in [79]. There were at least three biological replicates for each assay.

### 4.6. Stomatal Density Measurement

The third leaf at a consistent position of 20-day-old test-tube plantlets was collected from DK2, DK4, and DK7 transgenic lines and WT. Then, a sharp blade was used to cut and harvest fresh leaf blocks at the targeted area of the leaf within 1–3 min, where the area of the leaf block should be no more than 3 mm^2^. The target side of blocks (the side we wanted to observe) was labeled and immediately incubated with electron microscopy fixing solution (Servicebio) for 2 h at room temperature, then transferred into 4 °C for preservation. After that, leaf blocks were washed 3 times with 0.1 MPB (pH 7.4), for 15 min each, then transferred into 1% OsO_4_ in 0.1 MPB (pH 7.4) for 1–2 h at room temperature, and tissue blocks were washed 3 times in 0.1 M PB (pH 7.4), for 15 min each. Then, the plant blocks were dehydrated with various concentrations of ethanol. Later, specimens were attached to metallic stubs using carbon stickers and sputter-coated with gold for 30 s. Finally, the stomatal density was observed and images were taken with a scanning electron microscope (HITACHI, SU8100). There were three biological replicates for each process.

### 4.7. Prediction and Validation of stu-miR827a-5p Target Genes

Stu-miR827-5p target genes in potato were predicted using psRNATarget (http://plantgrn.noble.org/psRNAtarget/) (2017 Update) in potato [80]. The 21 nt mature sequence of stu-miR827-5p was submitted against the potato transcripts (Solanum tuberosum EST StGI-12.0/13.0) for target prediction. Among the predicted targets of stu-miR827-5p, a WRKY transcription factor gene (Soltu.DM.01G019140.1) was obtained (Table 2). The protein sequence of the target gene (Soltu.DM.01G019140.1) was used to identify conserved motifs in the NCBI Conserved Domain Database.

To validate the predicted target gene (*StWRKY48*) of stu-miR827-5p in potato, the modified RLM-5′RACE was performed using the GeneRacer Kit (Invitrogen). Total RNA was extracted from wildtype potato and directly ligated with the 5′RACE adapter, and then subsequently transcribed to cDNA using the reverse primers. The nested RACE-PCR was conducted to obtain the 5′ends of *StWRKY48* with the gene-specific primer and the GeneRacer 5′-adapter-specific primer (Table 4). The PCR fragments were then inserted into the pMD19-T vector, and individual clones were used for sequencing to identify the cleavage sites of *StWRKY48* mRNA.

### 4.8. Subcellular Localization Assay

The sequence of StWRKY48 was cloned from potato and then constructed into in the pSAK277 vector under the N-terminal of green fluorescent protein (GFP) from the fusion vector 35S::StWRKY48-GFP. The pSAK277 vector fused with GFP (35S-GFP) was used as the control. Those two constructs were transfected separately into *Agrobacterium tumefaciens* GV3101 and then were injected into the tobacco (*Nicotiana benthamiana*) epidermal cells according to a previous method [81]. After the transformed leaves were incubated for 48 h in the greenhouse, the plant epidermis was peeled off. Finally, GFP fluorescence was examined with confocal laser scanning microscopy (FV300 Fluoview OLYMPUS JAPAN CO., LTD, Tokyo, Japan).

### 4.9. Statistical Analysis

The variances of experimental data were processed with SPSS Statistics version 17.0 (SPSS Inc., Chicago, IL, USA). The data were presented as the mean ± standard error (SE).

## 5. Conclusions

In conclusion, we identified that stu-miR827-5p directly targets *StWRKY48* in potato, and cleavage positions of *StWRKY48* were effectively confirmed by the 5′RACE assay. Expression analysis also showed that stu-miR827-5p and *StWRKY48* exhibited an opposite expression pattern under drought stress. Suppressing the expression of stu-miR827-5p improved the drought tolerance of potato and led to a significant increase in leaf stomatal density compared to WT plants. In addition, the transcript levels of *StSDD1* and *StTMM* genes were significantly downregulated in transgenic lines. These results displayed that decreased expression of stu-miR827-5p might lead to overexpression of *StMAPK48*, which may contribute to regulating the drought adaptation of potato plants by regulating stomatal density.

## Figures and Tables

**Table 1:** The detailed information of stu-miR827.

miRNA	stu-miR827
MS	UUUGUUGAUGGUCAUCUAUUC
Gene ID	MI0025949
PS	UUUGUUGAUGGUCAUCUAUUCAUCAUAUCAUUUGGCAUAGUUUUUGCAACCAUUAAUAUGCCAUGAUUAGAUGAACAUCAACAAACA
LP (nt)	87
Families	miR827
NM (nt)	2
LM (nt)	21
Side	5
MFEs (kcal/mol)	−32.05
Chromosome	12
GenBank	CP047558.1

MS, mature sequences; PS, precursor sequences; LS, length of sequences; NM, number of mismatches; LM, length of mature miRNAs; LP, length of precursor; MFEs, the free energy of the thermodynamic ensemble.

**Table 2:** Predicted target gene of stu-miR827.

miRNA	miRNA Sequence	Target Gene ID	Target Annotations
stu-miR827	UUUGUUGAUGGUCAUCUAUUC	Soltu.DM.01G019140.1	*StWRKY48*

**Table 3:** Oligonucleotides used for RT-qPCR analysis.

Gene	Oligonucleotides Forward (F) and Reverse (R)	Amplicon Length (bp)
*Stu-miR827*	F 5′-TGCCTGGCTCCCTGTATGCCA-3′	-
*St18sRNA*	R 5′-TTAGAGGAAGGAGAAGTCGTAACAA-3′	-
*StWRKY48*	F 5′-TGCTACCACCAAGTTTCCA -3′	171
	R 5′-CCGAAGAGTAAGAACCGAGG -3′	
*StSDD1*	F 5′-TGAAGGAGAGCCTGTCATAAG -3′	111
	R 5′-GGCATAGGATGTAACCGATT -3′	
*StEPF*	F 5′-AAGACAACATTGGCAAGGG -3′	169
	R 5′-GCAACAGGACAAGTCTCAGC -3′	
*StTMM*	F 5′-GAAATAATGGAGGTGAGCGT -3′	137
	R 5′-GTTGCTAAACGATGCCTTG -3′	
*StSPCH*	F 5′-GACCATCTATCTGTGCTACGC -3′	130
	R 5′-TTGGCTTCTAAGGATTGGAG -3′	
*StMUTE*	F 5′-CGAAAGAGCCTAAGCCCTA -3′	130
	R 5′-AACATCAGCCACTGGAGAA -3′	
*StFAMA*	F 5′-TGAGGAAGTTGAGAGCCAA -3′	118
	R 5′-CCCTTTGAACATAGGAGCC -3′	
*StEF1α*	F 5′- ATTGGAAACGGATATGCTCCA-3′	101
	R 5′- TCCTTACCTGAACGCCTGTCA-3′	

**Table 4:** The nested PCR primers for the miRNA cleavage sites’ validation by RLM-5′ RACE.

Target Gene	Genomic Locust	Outer Primer	Inner Primer	Size
*StWRKY48*	Soltu.DM.01G019140.1	CAGGCTATCAAATGCCGATGA	TGAGCAGAAAGGCAGCATTCAAGACAATC	397 bp

## Supplementary Materials

The following supporting information can be downloaded in https://www.mdpi.com/article/10.3390/ijms232314805/s1.
