# Supplementary material for: Stu-miR827-Targeted StWRKY48 Transcription Factor Negatively Regulates Drought Tolerance of Potato by Increasing Leaf Stomatal Density
Source: Int J Mol Sci. 2022 Nov 26;23(23):14805. doi: 10.3390/ijms232314805 (PMC9741430; doi:10.3390/ijms232314805)
Supplement: Supplementary file 1 [file ijms-23-14805-s001.zip › ijms-2016901-supplementary.pdf]

Table S1. The basic properties and characteristics of *StWRKY48*.

**Gene**	***StWRKY48***
Chr	1
Genomic Coords (5'-3')	Soltu.DM.01G019140
Position	52037832 - 52035935
CDS (bp)	1,020
Exons (No)	3
Full length (bp)	1,481
Amino acid (aa)	339
pI	9.22
MW (kDa)	67,184.24
